# Quantitative Analysis of Broken Rotor Bars in Cage Motor Based on Energy Characteristics of Vibration Signals

**DOI:** 10.1155/2022/9312876

**Published:** 2022-06-03

**Authors:** Jie Shi, Haifeng Shen, Zhenkai Ding

**Affiliations:** School of Electronics and Information Engineering, Suzhou University of Science and Technology, Suzhou, Jiangsu Province, China

## Abstract

The rotor, as the power output device of a cage motor, is subject to a type of invisible fault, BRB, during long-term use. The conventional motor vibration signal fault monitoring system only analyzes the rotor qualitatively for the fault of BRBs and cannot evaluate the fault degree of BRBs quantitatively. Moreover, the vibration signal used for monitoring has nonstationary and nonlinear characteristics. It is necessary to manually determine the time window and basis function when extracting the characteristics of the time-frequency domain. To address these problems, this paper proposes a method for quantitative analysis of BRBs based on CEEMD decomposition and weight transformation for feature extraction and then uses the AdaBoost to construct a classifier. The method applies CEEMD for adaptive decomposition while extracting IMFs' energy as the initial feature values, uses OOB for contribution evaluation of features to construct weight vectors, and performs a spatial transformation on the original feature values to expand the differences between the feature vectors. To verify the effectiveness and superiority of the method, vibration signals were collected from motors in four BRB states to produce rotor fault data sets in this paper. The experiment results show that the feature extraction method based on CEEMD decomposition and weight transformation can better extract the feature vectors from the vibration signals, and the constructed classifier can accurately perform quantitative analysis of BRB fault.

## 1. Introduction

Induction motors are widely used in industrial production because of their economical, reliable, and ease of control. In the course of their use, they are subject to mechanical, thermal, and electrical stresses as well as environmental factors, which inevitably lead to failures [1]. The induction motors are subject to a wide range of failures. Among the various possible failures of induction motors, a broken rotor bar (BRB) is a kind of hidden fault [2]. This means that a single or small number of broken rotor bars do not have a noticeable effect on the function of the motor, as the current that should flow from the broken bar can be dispersed to the adjacent bars, thus ensuring normal motor operation. However, if they are not repaired at an early stage of failure, this can lead to excessive vibration, energy loss, and other problems. Also, under electrical and thermal stress, the number of BRB expands, and a sweeping failure may occur under centrifugal force, leading to motor shutdown and even causing injury to personnel and machinery.

Unlike the design for the stator, the design and manufacture of cage rotors have remained virtually unchanged for many years, and rotor failures now account for about 5–10% of all induction motor failures [3]. There are two types of cage rotors: cast and fabricated. Previously, cast rotors were only used on small machines. With the advent of cast ducted rotors, cast rotors can even be used for motors under the 3,000 kW, and the field of application of cage motors continues to grow [4]. Therefore, the monitoring of rotor health and the quantitative analysis of BRB faults in motors in using is a very important and challenging task.

Several condition monitoring techniques have been developed to monitor the health of motors, including air gap torque monitoring, noise measurement, thermal monitoring, partial discharge measurement, instantaneous angular velocity, instantaneous power, surge testing, vibration monitoring, and current monitoring [5]. Vibration monitoring and noise monitoring have been extensively investigated for their ability to detect both electrical and mechanical faults. When a fault occurs, the vibration characteristics of the machine will change [6]. Fault diagnosis based on vibration signals is a well-established field covering a wide range of techniques, mainly using time, frequency, and time-frequency feature extraction. Acoustic noise monitoring is performed by investigating the noise spectrum, and the acoustic signal shows the acoustic stress waves emerging from the energy release due to microstructural changes in the material or structure of the motor in different states [7], whereas noise monitoring techniques are sensitive to environmental noise during use, filtering, signal identification, and noise reduction become critical at the pre-processing stage, resulting in the technique being far less effective than laboratory results during application, so this monitoring technique is often used as a secondary monitoring technique [8].

As a multifactor coupled rotating system, the vibration signal is a good representation of the motor's operating conditions under different faults. When a motor has a BRB fault, the vibration signal will produce torque pulsations and speed oscillations [9]. At present, the research work on BRB faults feature recognition is mainly based on this. Sabbaghian-Bidgoli and Poshtan combined the wavelet packet decomposition (WPT) and the Hilbert transform to propose the Hilbert–Huang transform (HHT) to detect the characteristic frequency of the fault [10]. The results show that this hybrid technique exhibits better performance than the conventional wavelet packet transform. Moon and Dae used the finite element method to analyze the effect of vibration on motor life and performance due to thermal deformation of the frame caused by heat during motor operation [11]. Liu et al. used empirical mode decomposition (EMD) thresholding to denoise the vibration signals and applied probabilistic neural networks (PNN) to classify them [12]. The signal is decomposed by ensemble empirical mode decomposition (EEMD) with curve coding techniques to achieve stable identification of motor faults [13]. Ke et al. used a genetic algorithm to optimize the white noise amplitude in EEMD for bearing fault diagnosis in electric motors [14]. Miceli et al. used time-frequency analysis methods to apply axial and radial vibration signals for BRB faults diagnosis [15]. Xiao et al. proposed a feature extraction method combining complete ensemble empirical mode decomposition (CEEMD) and composite multiscale basic scale entropy (CMBSE), which can extract weak signal features under strong noise background [16]. A specific Zhao-Atlas-Marks distribution was proposed by Climente-Alarcón et al. to extract the harmonic components associated with BRB faults [17]. All of these research works have achieved effective detection of BRB faults using vibration signals. The above methods solve the problem of fault diagnosis of motors in specific environments and are able to identify BRB faults in motors, but they are qualitative fault analysis and cannot achieve a quantitative diagnosis of breakage and rotor health discrimination, which is not conducive to the development of inspection and maintenance plans for continuously operating equipment.

The amplitude, frequency, and axial trajectory of the vibration signal can reflect the characteristics of the fault [18]. When using common methods such as correlation analysis and Fourier transform for signal feature mining, the analysis and processing of nonlinear nonperiodic signals can only extract features in the time or frequency domain, resulting in a large amount of information being lost. The torque output of the motor system is often affected by load changes, which makes nonstationary and nonlinear characteristics of vibration signal more significant [19]. The wavelet transform can be used to improve this phenomenon through the choice of the time window in signal analysis, but how to determine the appropriate wavelet basis and the number of decomposition layers is an important issue. On the other hand, the wavelet bases are explicitly chosen in the wavelet transform process and cannot be adaptively adjusted during the decomposition process according to the signal characteristics [20]. Unlike the wavelet transform, empirical mode decomposition is an adaptive processing technique that can be applied to the analysis of complex signals based on the inherent characteristics of the signal [21]. Although it avoids the choice of decomposition layers and wavelet bases and has multiresolution analysis capability, while there are sudden changes or disturbances in the signal, part of the timescale will be lost, leading to a severe modal mixing phenomenon [22]. As an important problem in EMD, the improvement method of modal mixing has been one of the current research hotspots. Among the many improvement algorithms, EEMD is a noise-friendly method, known for its ability to suppress modal mixing, but its noise suppression mechanism will produce great residual noise, which creates obstacles for further feature extraction [23, 24].

To address the above problems and, considering the complexity of the rotor fault signal, to improve the stability of feature extraction and ensure the accuracy of quantitative analysis, this paper starts from the vibration signal of the motor, extracts the energy parameters of different layers by adaptive decomposition through CEEMD, and adjusts the recognition weights of each layer of energy through the random forest out-of-bag (OOB) estimation method. Finally, An AdaBoost strong classifier is used to quantitatively identify the BRB faults.

## 2. Complete Ensemble Empirical Mode Decomposition

### 2.1. Empirical Mode Decomposition

Empirical mode decomposition is an adaptive algorithm that decomposes a signal into a series of intrinsic mode functions (IMFs) based on the characteristics of the signal itself and is particularly suitable for the analysis of nonlinear and nonstationary signals. The decomposition process is only based on the original signal data, and the IMFs are separated layer by layer from high to low according to the oscillation law of the signal. Finally, the remaining component with the longest oscillation period is regarded as the residual. The IMFs should satisfy two conditions: (1) the number of extremes and the number of crossing zeros must be equal or differ by at most once, and (2) at any point, the envelope defined by the local maximum and the envelope defined by the local minimum have an average value of zero.

For a given signal *x*(*t*), the EMD method first extracts the local extremes to form the upper and lower envelopes and then interpolates the local maximum and local minimum, respectively. The average of the upper and lower envelopes is then calculated at *m*(*t*), and the average is subtracted from the signal using *h*(*t*)=*x*(*t*) − *m*(*t*). The signal *x*(*t*) is replaced by *h*(*t*), and the above two steps are repeated until *h*(*t*) satisfies the two conditions of the IMFs; then the process is completed. The residual *r*(*t*) is defined as *r*(*t*)=*x*(*t*) − *h*(*t*) and considered as a new signal continuously decomposed by the above steps. If the residual satisfies the stopping criteria, the decomposition process ends. The original signal *x*(*t*) will eventually be decomposed into multiple IMFs and a final residual *r*(*t*) as shown in the following equation:

(1) $$\begin{array}{c} x\left(t\right)=r\left(t\right)+\sum_{i=1}^{n}IMF_{i}\left(t\right). \end{array}$$

Although the EMD can be adaptively adjusted to the inherent characteristics of the signal, it has two important drawbacks: (1) the overshoot or undershoot of the spline fitting method may lead to large errors, which in turn affect the structure of the IMFs, and (2) if there are sudden changes or perturbations in the signal, the EMD will lose some of its timescale, which in turn will cause severe modal mixing.

### 2.2. Ensemble Empirical Mode Decomposition

The ensemble empirical mode decomposition algorithm is an improved algorithm that effectively solves the modal mixing problem in EMD. The algorithm adds *N* nonrepeating Gaussian white noises of rank *L* to the original signal and employs EMD to modal decompose the noise-added signal, taking the average value of the corresponding IMFs as the final result. The addition of white noise enriches the spectral content of the signal and can effectively prevent modal mixing. The algorithm assumes that there is a sufficient number of noise additions to ensure that the final noise energy is zeroed when the signal is reconstructed, but in practice, due to the limitation of computing resources, the number of noise additions is often insufficient to achieve noise cancellation, making the IMFs contain residual noise. At the same time, the introduction of noise breaks the energy distribution between the layers of the original signal, making the fault features extracted from the IMFs no longer reliable.

### 2.3. Complete Ensemble Empirical Mode Decomposition

To address the problems in the application of EMD and EEMD, Colominas et al. proposed a complete ensemble empirical mode decomposition (CEEMD) [25]. The method adds noise to each decomposition stage and uses the original signal minus the noise to construct the complementary signal, canceling out the problem of noise present in the signal by taking the mean of the two signals' EMD. Not only does it solve the mode mixing problem, but it also provides an accurate reconstruction of the original signal. The operational steps are as follows:

Step 1. Generate a random Gaussian white noise *n*(*t*) with a noise level of *L* and construct a noise-added signal *s*_*a*_(*t*) and its complementary signal *s*_*b*_(*t*):
  (2) $$\begin{array}{c} \left\{\begin{array}{c} s_{a}\left(t\right)=x\left(t\right)+n\left(t\right),\\ s_{b}\left(t\right)=x\left(t\right)-n\left(t\right). \end{array}\right. \end{array}$$
Step 2. Decompose *s*_*a*_(*t*) and *s*_*b*_(*t*) with EMD to get the layers *IMF*_*a*_^*i*^ and *IMF*_*b*_^*i*^ and take the average value of both as *IMF*^*i*^:
  (3) $$\begin{array}{c} IMF^{i}=\frac{IMF_{a}^{i}+IMF_{b}^{i}}{2}. \end{array}$$
  Step 3. Repeat steps 1 and 2 *N* times but add a different white noise each time.
  Step 4. Calculate the average value of the corresponding IMFs as the final result. The original signal *x*(*t*) will be decomposed into the same form as the EMD, as shown in equation (1).

## 3. Feature Weight Transformation

### 3.1. Feature Extraction

The objective of feature extraction is to determine a feature vector that characterizes the variability between samples based on a given set of samples so that the samples can be correctly classified [26]. The vibration signal contains a large amount of information that characterizes the state of the motor rotor, from which a feature vector can be extracted for state classification. However, due to the complex nonsmooth, nonlinear nature of the vibration signal, although CEEMD has adaptively decomposed the signal into IMFs in different frequency domains, the features are still not obvious and cannot be directly used for quantitative analysis of the BRB fault. Therefore, we need to further extract the fault features from the IMFs.

The core idea of CEEMD decomposition is to eliminate modal mixing by adding white noise to the signal to be decomposed and to eliminate the effect of noise on the original signal by complementary signals. Therefore, IMF itself has antinoise characteristics, and there is no need for filtering operation in feature extraction. The IMFs are essentially a series of central envelopes of similar frequencies obtained by subtracting the signal to find the difference, characterizing the degree of oscillation between the different modes. The vibration signal of a motor has a strong structural response, and this vibration is an inherent property of the mechanical state of the motor. When the motor rotor changes in response to different BRB faults, the response of the vibration signal and the energy distribution between the IMFs will also change. By analyzing the energy distribution of IMF in each layer, the number of BRBS of the motor can be determined. Lu et al. used the Teager–Kaiser energy factor to analyze the main oscillation frequency of the stator harmonics to quantify the BRBs in a specific state, which indirectly demonstrates that the occurrence of BRBs affects the energy distribution between the IMFs of the motor vibration signal distribution [27].

The energy characteristic formula of the IMFs is shown in the following equation:

(4) $$\begin{array}{c} E_{i}=\frac{\sum_{j=1}^{M}\left|c_{j}\right|}{\sum_{1}^{k}\sum_{j=1}^{M}\left|c_{j}\right|}. \end{array}$$

Where *i*=1,2,…, *k*, *k* is the number of IMF components, *M* is the data length of the IMF component, *c*_*j*_ is the *j*-th data of the IMF component, and *E*_*i*_ is the proportion of the *i*-th IMF component in the total energy, and its value is taken in the interval [0, 1]. If *E*_*j*_ is used directly as the eigenvalue to form the eigenvector characterizing the state of the motor, this will lead to local stacking in the characterization space, making the eigenvectors with clear differences extremely similar. Therefore, the eigenvectors should first be logarithmically transformed with respect to each other to expand the characterization space of the eigenvectors. To further improve the ability of the feature vectors to characterize the state of BRB, this paper uses the random forest out-of-bag (OOB) estimation method to measure the contribution of features in state identification and uses this as a basis to transform the weights of the features to expand the interclass variation of the samples.

### 3.2. Feature Importance Metric

The random forest OOB estimation method is an integrated machine learning method using decision trees as base classifiers [28, 29]. Its training steps are as follows:

    Step 1. The training sample set and OOB data set are generated from the original sample set by the bootstrap resampling method
   Step 2. Train the decision tree as the base classifier using the training data set and test its OOB error using the OOB data set, denoted as *errOOB*_1_
    Step 3. Add noise to the feature Q of all samples in the OOB data set and calculate the OOB error of the decision tree again, denoted as *errOOB*_2_
   Step 4. Assuming there are *K* decision trees in the forest, the importance of feature *Q* is *Q*_*p*_=(*errOOB*_2_ − *errOOB*_1_)/*K*

*Q*_*p*_ is possible to characterize the importance of a feature because, if the accuracy of the OOB data drops significantly when random noise is added (i.e., *errOOB*_2_ goes up), this indicates that the feature has a significant impact on the prediction of the sample, which in turn indicates that it is of high importance. Each feature is judged in the random forest training process, with the most widely used measure of feature importance being “ranked importance.” This is based on the change in the classification error rate of the random forest model before and after a feature label is changed in the OOB data [30].

If a feature is used for classification and the results are less different from those of random classification, the contribution of this feature to the classification effect is considered to be minimal. Weakening these features empirically has little effect on classification accuracy. *Q*_*p*_ is a good measure of how well the features contribute to the classification effect of a sample, and using them as weights to scale and transform the feature values can expand the interclass differences or spatial distances of the samples and improve the effectiveness of the samples when classified or clustered. This is certainly a good way to deal with samples with small differences in feature vectors between classes.

## 4. AdaBoost Quantitative Fault Classifier

Integration learning is an important area of research in machine learning. Instead of trying to obtain a single optimal classifier, it trains a set of weak classifiers from existing data and then combines the weak classifiers into a strong classifier according to a certain strategy. AdaBoost is one of the representative algorithms of integrated learning [31], which can take a simple, coarse prediction method with low accuracy and construct a complex prediction method with high accuracy by specific rules [32]. The aim of this paper is to quantitatively analyze the BRB status of motor rotors. The task is achieved not only by simple qualitative analysis of faults but also by further classification of small difference samples, which cannot or is difficult to achieve with a single classifier alone. Therefore, we can construct multiple weak classifiers, use threshold control to discriminate whether the sample belongs to that small class, and integrate the weak classifiers into strong classifiers through strategy control to finally achieve accurate classification.

There are two main aspects of integration learning: the update strategy of sample weights and the combination strategy of base classifiers. The feature weight transformation using random forest OOB estimation described in Section 3.2 is a sample weight update strategy and can be considered as a pre-processing of samples. The use of decision trees as base classifiers can maintain the consistency of the classification system, and the steps for composing a strong classifier are as follows:

  Step 1. Given the data set *S*={(*X*_1_, *Y*_1_),…(*X*_*i*_, *Y*_*i*_),…, (*X*_*n*_, *Y*_*n*_)}, where *X*_*i*_=(*IMF*_1_′, *IMF*_2_′,…, *IMF*_8_′) is the feature vector, *Y*_*i*_ is the classification flag, and *n* is the number of training samples
  Step 2. Initialize the sample weight distribution vector for k iterations *D*_*k*_(*i*)=1/*n*
  Step 3. Train the base classifier $T_{k}\left(X_{i}\right)=\arg\underset{T}{\min}\sum_{t=1}^{k}D_{k}\left(i\right)\left[T_{k}\left(X_{i}\right)\neq Y_{i}\right]$ and its classification error rate *ε*_*k*_=∑_*i*=1_^*n*^*D*_*t*_(*i*)[*T*_*k*_(*X*_*i*_) ≠ *Y*_*i*_], where [*T*_*k*_(*X*_*i*_) ≠ *Y*_*i*_] is the indicator function that takes on a value of 1 when *T*_*k*_(*X*_*i*_) ≠ *Y*_*i*_ and 0 otherwise
  Step 4. Calculate the weights of the base classifier *α*_*k*_=1/2ln1 − *ε*_*k*_/*ε*_*k*_
  Step 5. Update the sample weights *D*_*k*+1_(*i*)=*D*_*k*_(*i*)^−*α*_*i*_^/∑_*k*=1_^*m*^*D*_*k*_(*i*) to obtain the strong classifier *H*(*X*)=sign(∑_*t*=1_^*k*^*α*_*k*_*T*_*k*_(*X*))

Although the AdaBoost classification algorithm has a certain sample weight update mechanism, a large number of base classifiers are required to support a reliable classification effect. Using out-of-bag estimation to pre-process the data according to the importance of weight transformation can reduce the number of base classifiers required by the quantitative classifier and improve the discriminative efficiency of the model.

## 5. BRB Quantitative Analysis

**Figure 1:**
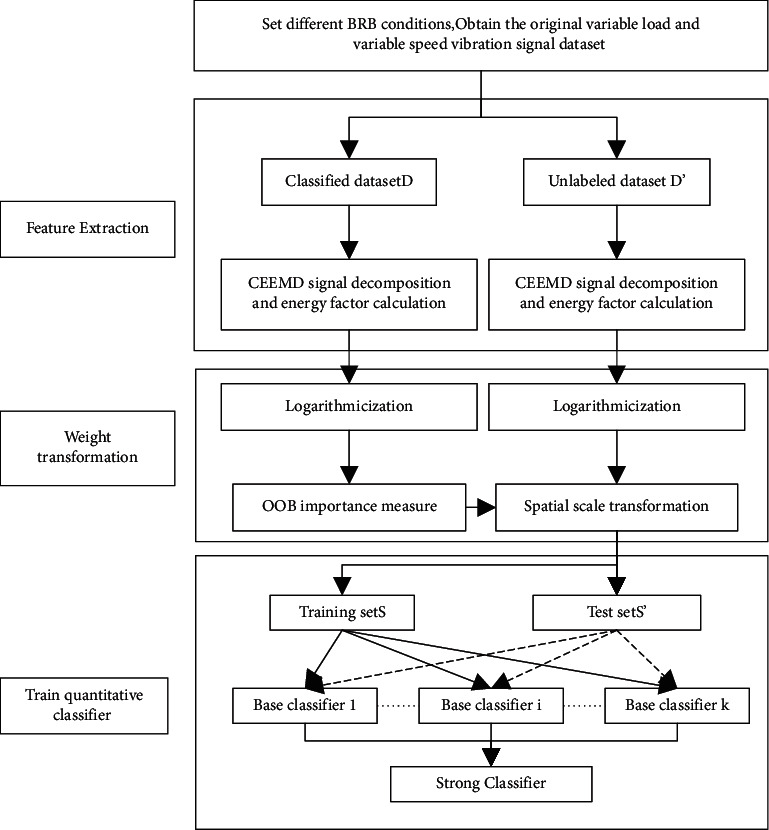
Flow chart for vibration signal analysis.

The flow of quantitative analysis of BRB faults in cage motor rotors based on the energy characteristics of vibration signals is shown in Figure 1. The process is divided into three main stages, namely feature extraction, weight transformation, and classifier training.

In the first stage, by manually setting the operating state of the fault simulation motor and performing three times mean filtering during the signal acquisition process, the motor vibration signals in different states are obtained and decomposed using CEEMD to obtain a series of IMFs, and the energy factor of each layer is calculated as the primary feature for quantitative rotor analysis.

The second stage logarithm the primary features and uses OOB estimation to obtain a weight vector for the feature space scale transformation using the OOB estimation method for the feature importance measure on the annotated data sets.

The third stage is the training of the AdaBoost rotor broken bar quantitative classifier, where *k* base classifiers are trained from the training set to combine into a strong classifier, and the performance is evaluated using the test set.

## 6. Experimental Analysis and Discussion

**Figure 2:**
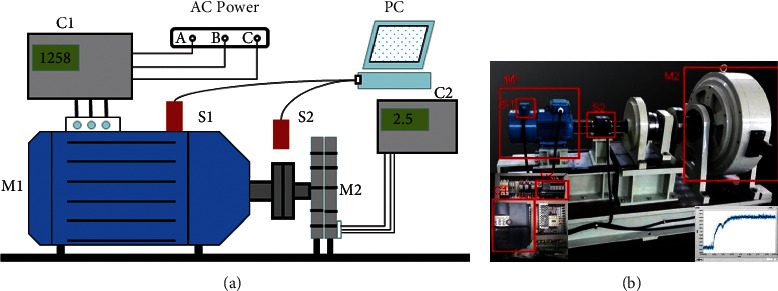
Signal acquisition system for broken rotor bars in cage motors: (a) sketch map and (b) physical map.

Figure 2 shows a cage motor rotor vibration signal acquisition system consisting of M1, three-phase cage asynchronous motor; M2, magnetic powder brake; C1, inverter; C2, tension controller; S1, accelerometer; S2, photoelectric tachometer; and a computer. The experiments simulate 0–3 BRBs states by manually punching damage to the motor rotor. In order to obtain the vibration signals of the motor in different states, in addition to setting the corresponding faults, the vibration signals under variable load and variable speed operation are obtained by controlling the tension controller and the frequency converter, respectively.

### 6.1. CEEMD Performance Analysis

In order to verify the advantages of CEEMD over EMD and EEMD in signal decomposition, five sets of actual collected motor BRB vibration signals were decomposed, and the root mean square error (RMSE), mean absolute error (MAE), and Nash–Sutcliffe efficiency coefficient (NSE) are the evaluation indexes of the decomposition accuracy, and the three error evaluation indexes were calculated as follows:

(5) $$\begin{array}{c} \mathrm{RMSE}=\sqrt{\frac{1}{n}\sum_{i=1}^{n}{\left(y-y_{i}\right)}^{2}},\\ \mathrm{MAE}=\frac{1}{n}\sum_{i=1}^{n}\left|y-y_{i}\right|,\\ \mathrm{NSE}=1-\frac{\sum_{i=1}^{n}{\left(y-y_{i}\right)}^{2}}{\sum_{i=1}^{n}{\left(y-{\bar{y}}_{i}\right)}^{2}}, \end{array}$$

where *y* is the original value, *y*_*i*_ is the decomposition value, *n* is the number of original values, $\bar{y}$ is the average of *n* original values, and *NSE* ≤ 1, and the closer to 1, the higher the decomposition accuracy. The decomposition effects of EMD, EEMD, and CEEMD are shown in Table 1. Comparing the data performance of the three, it can be found that the decomposition error and algorithm stability of EEMD are the worst because EEMD adds random noise to the original signal, which is not completely controllable and the number of noise additions cannot be increased indefinitely to eliminate the influence of noise, while CEEMD also adds noise, but the noise energy is removed from the decomposition components through the signal complementary mechanism. The CEEMD also adds noise, but the noise energy is removed from the decomposition component by a signal complementary mechanism, which solves the modal mixing problem while perfectly maintaining the actual energy distribution of the signal.

Table 2 shows the data when using the OOB estimation method to sample the five subsets generated by the variable load data sets for the weight metric, with the sampling process being artificially set to have an unbalanced number of samples across the BRBs. The data in the table shows that the features have the same ranking in terms of their ability to characterize the state on different subsets and that the unbalanced data does not affect the ability of OOB to measure the importance of the features, while the standard deviation (SD) with minimal importance of each feature indicates not only the stability of the OOB algorithm but also the ability of the selected features to characterize the state of the motor rotor in a very stable manner.

### 6.2. Analysis of Variable Load Conditions

**Figure 3:**
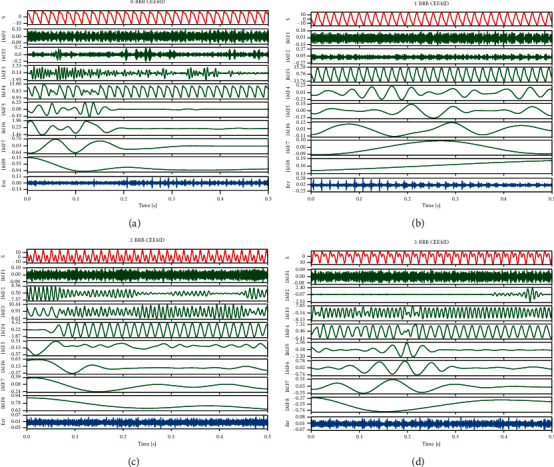
IMF under CEEMD of vibration signals for four rotor states under variable load conditions: (a) 0 BRB CEEMD decomposition results, (b) 1 BRB CEEMD decomposition results, (c) 2 BRB CEEMD decomposition results, and (d) 3 BRB CEEMD decomposition results.

A set of vibration signals in four states was randomly selected from the variable load data sets, and the four types of signals were first decomposed using CEEMD, and the IMFs were sorted from highest to lowest according to the signal frequencies, as shown in Figure 3, where *S* is the original signal, IMF is the decomposed modal function of each layer, and Err is the final decomposition residual. Although there is no significant difference between the original signal *S* at different BRB states, the difference in the energy distribution of the vibration signal at different BRBs can be directly observed by the decomposition of each IMF, and the lower the frequency the more obvious the difference.

**Figure 4:**
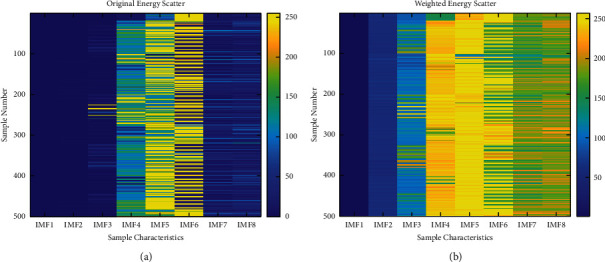
Energy distribution before and after IMF weighting for variable load conditions: (a) original IMF energy distribution and (b) weighted IMF energy distribution.

The energy features of the intrinsic modal functions of each layer are calculated as signal features and form the input feature vector. The importance of the feature vectors is calculated as weights after logarization to obtain the weight transform vectors of the feature values. The initial feature vectors are spatially transformed according to the weights to form the feature vectors used for state identification. The energy distribution of each IMF layer before and after weighting is shown in Figure 4. To increase visualization the energy distribution is associated with the [0, 255] thermal interval, where each row is a complete feature vector, 0–400 corresponds to 0–3 BRB, and each BRB subset has 100 samples. As shown in Figure 4(a), the original feature energy distribution is more extreme, with IMF1, IMF2, IMF3, IMF7, and IMF8 distributed in the 0–50 range, while IMF4, IMF5, and IMF6 occupy the 150–200 interval. The huge uneven distribution of features makes the intersample capability degrade, which will further lead to the failure of BRB quantitative analysis. In contrast, as shown in Figure 4(b), the feature vectors have been logarithmically and the weight redistributed by importance; the energy distribution is more even; and the intra- and interclass differences of the samples have been reflected.

Randomly select 0.5 s in the 1BRB data set and analyze according to the above process to obtain their feature weight, as shown in Table 3. From the table, it can be seen that for the vibration signal data sets collected at different time periods, the ranking of feature importance by weights is not affected by load changes and the feature importance measurement by weights has good stability.

Similarly, select time windows of 0.25 s, 0.5 s, 0.75 s, and 1 s, respectively, in the 1BRB data set. The analysis was carried out according to the above process, and their weights were derived as shown in Table 4, where the eigenvalues *x*_1_ ~ *x*_8_ are the energy characteristics of the IMF in descending order of frequency. As can be seen from the table, the ranking of the importance of the weights on the features did not change for vibration signals acquired in different length time windows. It can be seen that the extraction of fault features by CEEMD for signal decomposition is not affected by the time window, does not require manual determination of the basis function, and is highly adaptive.

**Figure 5:**
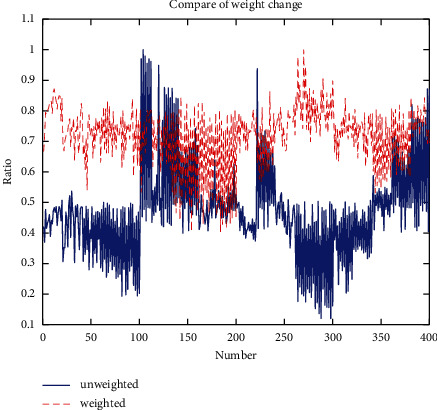
Comparison of sample distances before and after weight transformation for variable load conditions.

Based on the weights obtained in Tables 3 and 4 for different time periods and different time windows, the joint average of 0.17655, 0.16247, 0.12481, 0.13366, 0.12039, 0.09513, 0.08668, and 0.10030 were taken as the final weights determined by this method. The intra- and interclass differences of the feature vectors after the weighted transformation were further analyzed. Considering that the feature space corresponding to the weighted transformed feature vectors has been changed and there is scale variation, it is obviously inappropriate if the general Euclidean distance method is chosen to measure the spatial distance between samples at this point. Therefore, we select the vector mode of *Xs* = [1, 1, 1, 1, 1, 1, 1, 1] as the base in the original feature space and the vector mode of *Xs*′ = [0.17655, 0.16247, 0.12481, 0.13366, 0.12039, 0.09513, 0.08668, 0.10030] as the base in the transformed feature space and use the normalized ratio between the vector mode of the feature vectors and the base in each space as the index to measure the effect of the spatial transformation. The normalized ratios before and after the weighting transformation are shown in Figure 5. The overall improvement of the vector modes of the weighted feature vectors with respect to the baseline vectors is evident, and the original interclass differences are still maintained, which indicates that the weighting transformation can achieve a uniform distribution of features without breaking the expression ability of the features.

Traditional classification algorithms use a supervised learning model that relies on the manual selection of the sample data set. To demonstrate the ability of feature extraction to characterize the samples, this paper uses unsupervised learning to cluster the feature vectors that have undergone a weight weighting transformation by different clustering algorithms with the clustering accuracy shown in Table 5 for the CEEMD weighting transformation. In order to further verify the advanced nature of the proposed method, the fault characteristics extracted from CEEMD and discrete wavelet analysis without weight transformation are compared. According to the description of reference [27], the best parameters of the wavelet method are selected: the wavelet base is db3 and the number of decomposition layers is 8. The clustering accuracy of the features extracted by the different methods is shown in Table 5. The clustering effect of the CEEMD weight transform is the best in terms of both individual clustering algorithms and overall average accuracy, with an average improvement of 4.3% compared to the other methods. In contrast, the accuracy of the feature vectors extracted by the other methods was average, with a large difference in accuracy on different clustering algorithms, and even a failure. The failure phenomenon may occur because the CEEMD decomposition shows modal mixing and the wavelet bases of wavelet decomposition have inconsistent time windows under different signals. These problems lead to lopsided feature extraction, resulting in huge deviations in performance under different clustering algorithms.

**Figure 6:**
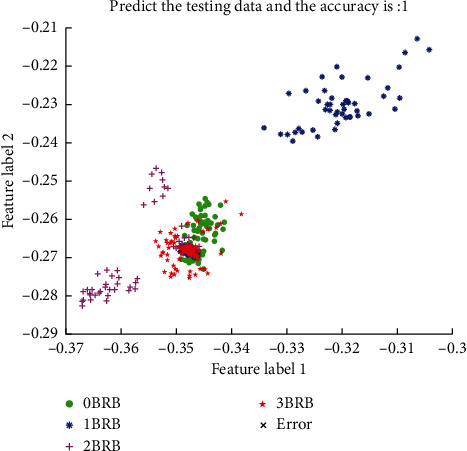
Classification effect of the AadaBoost broken bar quantitative classifier for variable load conditions.

The ultimate aim of feature weight transformation is to enable better quantitative analysis of rotor break bars. The unsupervised learning approach, although good at analyzing intra- and interclass differences in samples, has the problem of not being able to quantify the effect in practical engineering use. Supervised learning is therefore required for model evaluation and application of classification models to practical engineering.  Figure 6 shows the test results of training a classification model using a ratio of 7:3 to divide the data set to generate a training set and a test set. The spatial distribution of the four different samples can be seen in the figure, and although there is still some interclass coupling between the 0 BRB and 3 BRB samples, the classifier still achieves an accuracy of 100%.

### 6.3. Analysis of Variable Speed Working Conditions

**Figure 7:**
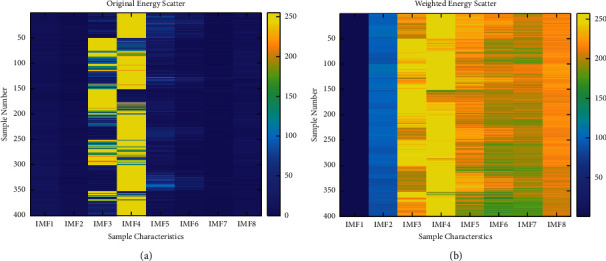
Energy distribution before and after IMF weighting for variable speed conditions: (a) raw IMF energy distribution and (b) weighted IMF energy distribution.

**Figure 8:**
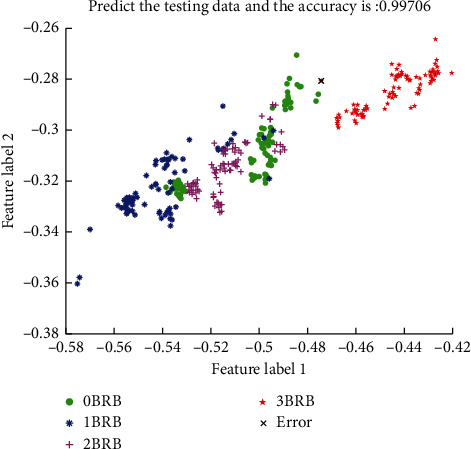
Classification effect of AadaBoost broken bar quantitative classifier for variable speed conditions.

The data sets collected under variable speed conditions were analyzed following the process in Section 6.2. Its weighted vector obtained by OOB estimation is [0.38491275, 0.19737257, 0.071427953, 0.082986883, 0.07176596, 0.072956693, 0.063359313, 0.055217877], and the weighted pre- and post-IMF interlayer energy distribution is shown in Figure 7. The results of the AdaBoost quantitative classifier are shown in Figure 8. The coupling between the 0 BRB, 1 BRB, and 2 BRB samples is still present, but the coupling is significantly lower compared to the data under variable torque conditions, and the classification accuracy is 99.7%.

The results of the above analysis show that the method proposed in this paper is able to identify broken strips and quantify the number of broken strips accurately under different operating modes of the motor.

## 7. Summary

This paper proposes a fault feature extraction method that uses CEEMD to decompose the vibration signal, uses the IMF energy factor of each layer as the initial feature, and uses the OOB estimation error to measure the contribution of the feature to the sample classification to construct the feature importance vector and further expands the differentiation between features by logarithmization and weight transformation. The feasibility of using this method and the high discrimination in fault feature extraction is verified based on the experimental data set, and the higher accuracy of this method is demonstrated by comparing it with existing feature extraction methods. Finally, an AdaBoost integrated learning method is used to construct a quantitative classifier for the number of broken rotor bars to achieve accurate identification of the number of broken rotor bars. The following conclusions can be drawn:

1. In this paper, the CEEMD decomposition technique is successfully applied to the fault feature extraction of electric motors, solving the previous problems of manually determining the time window and basis function when feature extraction is performed through the time-frequency domain, as well as the problem of residual noise introduced by EEMD during decomposition to destroy the energy distribution.
2. In this paper, the concept of weight transformation is proposed to measure the importance of eigenvalues in characterizing the rotor state of a motor, and it is applied to the spatial transformation of eigenvectors to improve the discrimination between different eigenvectors.
3. The quantitative classifier of broken rotor strips constructed by the AdaBoost method in this paper can achieve an accurate quantitative analysis of the number of broken rotor strips, which can be used by engineers to grasp the degree of rotor failure in time and reasonably arrange the inspection and maintenance schedule.

## 8. Feature Work

The solution proposed in this paper is only capable of quantitative analysis of faults with broken rotor strips. The identification and quantitative analysis of faults such as stator insulation damage, bearing wear, and rotor eccentricity are not yet possible, which will be a further research direction for us. In addition, the current solution is based on the analysis of existing samples and cannot identify faults outside the data set, and it will be a challenging and meaningful task to achieve a monitoring system that grows to learn from unknown faults.

## Figures and Tables

**Table 1:** Comparison of the decomposition effects of EMD, EEMD, and CEEMD.

	No.	RMSE	MAE	NSE
EMD	1	0.04251	0.03330	0.99995
	2	0.03790	0.02887	0.99997
	3	0.04737	0.03955	0.99995
	4	0.04325	0.03504	0.99993
	5	0.06053	0.04897	0.99994
	Standard deviation	0.00862	0.00763	1.48*E* − 5

EEMD	1	0.37228	0.36575	0.99624
	2	0.40699	0.39852	0.99628
	3	0.81135	0.80659	0.98661
	4	0.29647	0.28544	0.99681
	5	1.48709	1.48375	0.96350
	Standard deviation	0.49619	0.498743	0.01428

CEEMD	1	0.01279	0.00944	1.0000
	2	0.01185	0.00907	1.0000
	3	0.01066	0.00822	1.0000
	4	0.01537	0.01112	0.9999
	5	0.01822	0.01275	0.9999
	Standard deviation	0.00302	0.00180	5.47*E* − 5

**Table 2:** Out-of-bag estimated feature weighting metrics.

No.	IMF1	IMF2	IMF3	IMF4	IMF5	IMF6	IMF7	IMF8
1	0.18293	0.16138	0.12307	0.12893	0.13046	0.10212	0.08357	0.08754
2	0.18618	0.16315	0.12426	0.12886	0.12973	0.10406	0.07993	0.08383
3	0.18623	0.15852	0.12397	0.12872	0.13185	0.10502	0.08087	0.08482
4	0.18976	0.16221	0.11840	0.12673	0.13012	0.10100	0.08440	0.08738
5	0.19356	0.16351	0.12032	0.12678	0.12009	0.09960	0.08670	0.08944
SD	0.00406	0.00199	0.00254	0.00114	0.00474	0.00221	0.00273	0.00226

**Table 3:** Weights corresponding to the feature values of the data set for different time periods.

Feature	*x*_1_	*x*_2_	*x*_3_	*x*_4_	*x*_5_	*x*_6_	*x*_7_	*x*_8_
0.5 s Data set 1	0.18196	0.16391	0.12252	0.12918	0.11843	0.09120	0.08841	0.10439
0.5 s Data set 2	0.17606	0.16068	0.12002	0.14404	0.11226	0.09302	0.08957	0.10435
0.5 s Data set 3	0.17056	0.16008	0.12291	0.14097	0.12642	0.09119	0.08708	0.10078

**Table 4:** Weights corresponding to the eigenvalues of the data set under different time windows.

Feature (s)	*x*_1_	*x*_2_	*x*_3_	*x*_4_	*x*_5_	*x*_6_	*x*_7_	*x*_8_
0.25	0.16635	0.16490	0.13442	0.13192	0.11311	0.10632	0.07856	0.10441
0.5	0.18196	0.16391	0.12252	0.12918	0.11843	0.09120	0.08841	0.10439
0.75	0.17617	0.16245	0.12817	0.13147	0.12357	0.09089	0.09111	0.09617
1	0.18277	0.16139	0.12311	0.12889	0.13053	0.10208	0.08363	0.08762

**Table 5:** Clustering accuracy of features extracted by different methods.

Clustering methods	CEEMD weighting transformation (%)	CEEMD (%)	Wavelet method (%)
*K*-means	37	36.75	36.75
Mean-shift	24.5	20.5	25
AGG	35.25	24.5	20.5
GMM	35	30.75	30.75
Spectral clustering	34.75	32.5	32
Average	33.3	29	29

## Data Availability

The data used to support the findings of this study are available from the corresponding author upon request.
